# A Forward Genetic Screen for Suppressors of Somatic P Granules in *Caenorhabditis elegans*

**DOI:** 10.1534/g3.115.019257

**Published:** 2015-06-22

**Authors:** Ashley L. Kelly, Michael J. Senter-Zapata, Anne C. Campbell, Hannah E. Lust, Monique E. Theriault, Karolina M. Andralojc, Dustin L. Updike

**Affiliations:** Kathryn W. Davis Center for Regenerative Biology and Medicine, Mount Desert Island Biological Laboratory, Bar Harbor, Maine 04672

**Keywords:** *C. elegans*, P granules, retinoblastoma, germline, synMuv B

## Abstract

In *Caenorhabditis elegans*, germline expression programs are actively repressed in somatic tissue by components of the synMuv (synthetic multi-vulva) B chromatin remodeling complex, which include homologs of tumor suppressors Retinoblastoma (Rb/LIN-*35*) and Malignant Brain Tumor (MBT/LIN-*61*). However, the full scope of pathways that suppress germline expression in the soma is unknown. To address this, we performed a mutagenesis and screened for somatic expression of GFP-tagged PGL-1, a core P-granule nucleating protein. Eight alleles were isolated from 4000 haploid genomes. Five of these alleles exhibit a synMuv phenotype, whereas the remaining three were identified as hypomorphic alleles of known synMuv B genes, *lin-13* and *dpl-1*. These findings suggest that most suppressors of germline programs in the soma of *C. elegans* are either required for viability or function through synMuv B chromatin regulation.

Cancer cells acquire a number of traits normally restricted to germline stem cells, including cellular immortality and the ability to self-renew. A subset of proteins exclusively found in the germ cells of the testis and/or ovary is overexpressed in most melanomas and is frequently found in breast, bladder, lung, and hepatocellular cancers (Sahin *et al.* 1998; Simpson *et al.* 2005; Whitehurst 2013). Germline-enriched proteins can be highly antigenic when expressed outside of the germline and are often associated with malignancy and poor patient prognosis (Simpson *et al.* 2005; Blanchard *et al.* 2013).

Research in *Caenorhabditis elegans* is providing much needed insight into how germline programs are repressed in the soma. One remarkable discovery is that components of the synMuv B chromatin remodeling complex, which include homologs of the tumor suppressors Retinoblastoma (Rb or LIN-35) and Malignant Brain Tumor (MBT or LIN-61), actively repress the somatic expression of germline-specific ribonucleoprotein aggregates called germ granules (Unhavaithaya *et al.* 2002; Wang *et al.* 2005; Cui *et al.* 2006; Petrella *et al.* 2011; Wu *et al.* 2012) . Germ granules are found in the germ-cell cytoplasm of many species, where they are central to the pluripotent and immortal potential of the germline (Strome and Updike 2015). In *C. elegans*, germ granules are called P granules, and when they are depleted both sperm-specific transcription and somatic differentiation are initiated in germ cells (Updike *et al.* 2014; Campbell and Updike 2015). Observations in various species suggest that the presence of germ granules outside of the germline could favor conditions that promote pluripotency and cell proliferation.

The somatic repression of germ-granule components by the synMuv B chromatin regulation complex is not exclusive to *C. elegans* (Georlette *et al.* 2007). In *Drosophila*, brain tumors in MBT mutants overexpress conserved germ-granule components like PIWI, VASA, and AUBERGINE, which are necessary for brain tumor formation in *mbt* mutant flies (Janic *et al.* 2010). Because of its promise in elucidating cancer signaling cofactors of Rb and MBT, the contribution of the synMuv B pathway to the repression of somatic P granules has been thoroughly investigated. However, it is still unclear how germ granules might promote conditions that favor oncogenesis, or whether other tumor-suppressor pathways, apart from synMuv B chromatin regulators, actively repress somatic germ-granule expression.

Through a genome-wide RNAi screen in *C. elegans*, we previously found several genes required to suppress somatic P-granule expression during embryogenesis and the first larval stage of development (Updike and Strome 2009). To determine if additional pathways in the soma suppress expression of germline programs, we took an unbiased approach using forward genetics to screen adult worms for ectopic P granules. Here we report that most, if not all, suppressors of germline programs in the soma are either required for viability or function through synMuv B chromatin regulation, and not some other pathway. We also describe new alleles of the synMuv B genes *lin-13* and *dpl-1* that express somatic P granules but do not readily exhibit a synMuv phenotype.

## Materials and Methods

### Strain maintenance

*C. elegans* strains were maintained as per standard protocols (Brenner 1974). TH206 [*pgl-1p*::*PGL-1*::*TY1*::*EGFP*::*3xFLAG + Cbr-unc-119(+)*]I, MT10430 *lin-35(n745)*I, and the CB4856 Hawaiian isolate were obtained from the Caenorhabditis Genetics Center (CGC). DUP10 [PGL-1::GFP]I; *lin-13(sam4)*III, DUP20 [PGL-1::GFP]I; *lin-13(sam12)*III, DUP21 [PGL-1::GFP]I; *dpl-1(sam13)*II, DUP6 [PGL-1::GFP]I; *sam1*, DUP14 [PGL-1::GFP]I; *sam8*, DUP15 [PGL-1::GFP]I; *sam9*, DUP16 [PGL-1::GFP]I; *sam10*, DUP25 [PGL-1::GFP]I; (*sam17*/+), DUP52 *samEx4(*WRM0614dE05 + pCFJ104*)*; [PGL-1::GFP]I; *dpl-1(sam13)*II, and DUP53 *samEx5*(WRM064aA06 + pCFJ104); [PGL-1::GFP]I; *lin-13(sam4)*III were generated in this study.

### Fosmid rescue

DUP10 was injected with the fosmid WRM064aA06 (20 ng/ul) to create DUP53, and DUP21 was injected with the fosmid WRM0614dE05 (20 ng/ul) to create DUP52. All injections used the *myo-3p*::mCherry coinjection marker pCFJ104 (10 ng/ul) (Frøkjaer-Jensen *et al.* 2008).

### Screen design

EMS mutagenesis was performed on TH206 worms using the standard protocol (Kutscher and Shaham 2014). Two thousand F1 progeny were cloned to individual plates, and F2 grandchildren were screened under a Leica M165FC fluorescence stereomicroscope for ectopic PGL-1::GFP during the larval and adult stages. Fluorescence images were captured and tiled on a Leica DMI6000B inverted scope using a 40× air objective.

### Mapping

CB4856 (Hawaiian) males were crossed into DUP10, DUP20, and DUP21 strains. F1 cross progeny were picked to new plates, and approximately 50 F2s with the somatic P-granule phenotype were selected from each cross. The progeny of these F2 animals were pooled and then whole genome–sequenced as previously described (Doitsidou *et al.* 2010). The three mutant strains (*sam4*, *sam12*, *sam13*) were multiplexed with nine additional mutants (unpublished), and all 12 samples were sequenced in a single lane on an Illumina HiSeq2500. The CloudMap pipeline was used to analyze mutant genome sequences, obtain map data, and find mutations as previously described (Minevich *et al.* 2012). The NCBI Sequence Read Archive is attached to BioProject #282736.

### Complementation

DUP10 males were crossed into DUP20 hermaphrodites, and male cross progeny were examined for somatic PGL-1::GFP expression. This is in contrast to DUP10 and DUP20 backcrossed to TH206, where PGL-1::GFP was constrained to the germline in all cross-progeny.

### RNAi feeding

RNAi feeding constructs were obtained from the Ahringer library (Kamath *et al.* 2003). The L4440 plasmid in HT115 bacteria was used as the RNAi control; RNAi experiments were performed at 20° unless otherwise stated. *lin-15a* RNAi for each strain was performed on L4 worms in three biological replicates and their progeny for Muv phenotypes were observed. To assay RNAi enhancement, three plates containing approximately 60 embryos each were placed on *his-44* RNAi feeding plates for each strain, and animals arrested during larval development were scored 2 d later. A *t*-test was used to calculate the significance of the enhancement compared to wild-type. To assay somatic P-granule suppression, L4s from DUP10, DUP20, DUP21, and TH206 were fed *mes-3*, *mes-4*, *mrg-1*, *lin-35*, *lin-61*, and control RNAi, and progeny were examined in the L3 and L4 larval stages. RNAi targets were blinded and three replicates (of 32 worms each) were quantified for somatic PGL-1::GFP expression in each strain. A *t*-test was used to calculate the significance of enhancement or suppression from control RNAi.

## Results and Discussion

**Figure 1:**
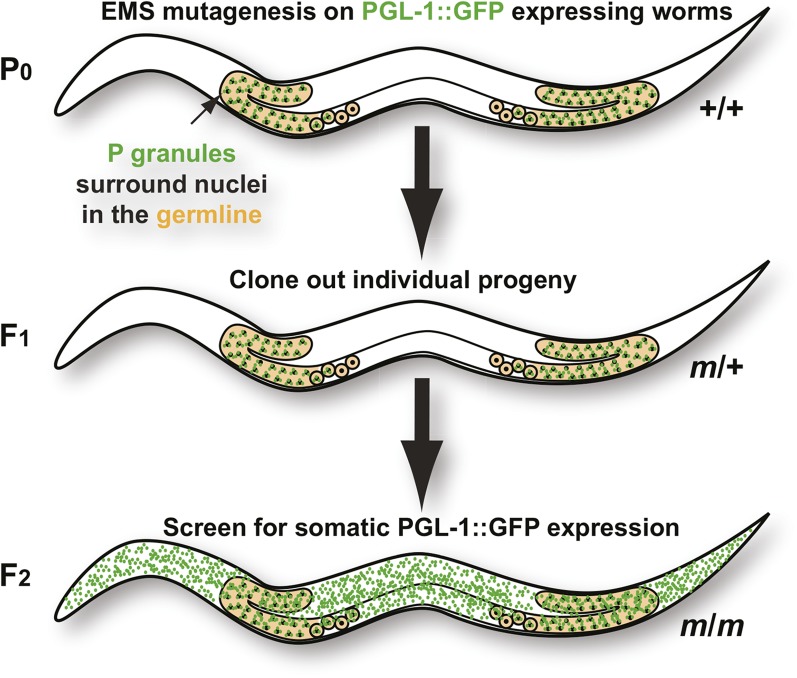
Screen for suppressors of somatic P-granule expression.

Forward genetic screens provide an unbiased approach to identifying the most significant players in a given biological pathway. To further elucidate the pathways that repress germline programs in the soma, EMS mutagenesis was performed on a *C. elegans* strain expressing the constitutive P-granule component, PGL-1, tagged with GFP. The F2 generation was then screened for somatic expression of PGL-1::GFP granules (Figure 1), and eight independent alleles were isolated (Table 1). Three of these exhibited intestinal PGL-1 granule expression, whereas five expressed PGL-1 granules throughout the soma.

**Table 1:** Somatic PGL-1::GFP alleles from the mutagenesis

Mutant Allele	Somatic P Granules	synMuv with *lin-15a* RNAi	Complementation
*sam1*	Whole worm	Yes	
*sam4*	Intestinal	No	*sam12*
*sam8*	Whole worm	Yes	
*sam9*	Intestinal	Yes	
*sam10*	Whole worm	Yes	
*sam12*	Whole worm	No	*sam4*
*sam13*	Intestinal	No	
*sam17*	Whole worm	Yes	

**Figure 2:**
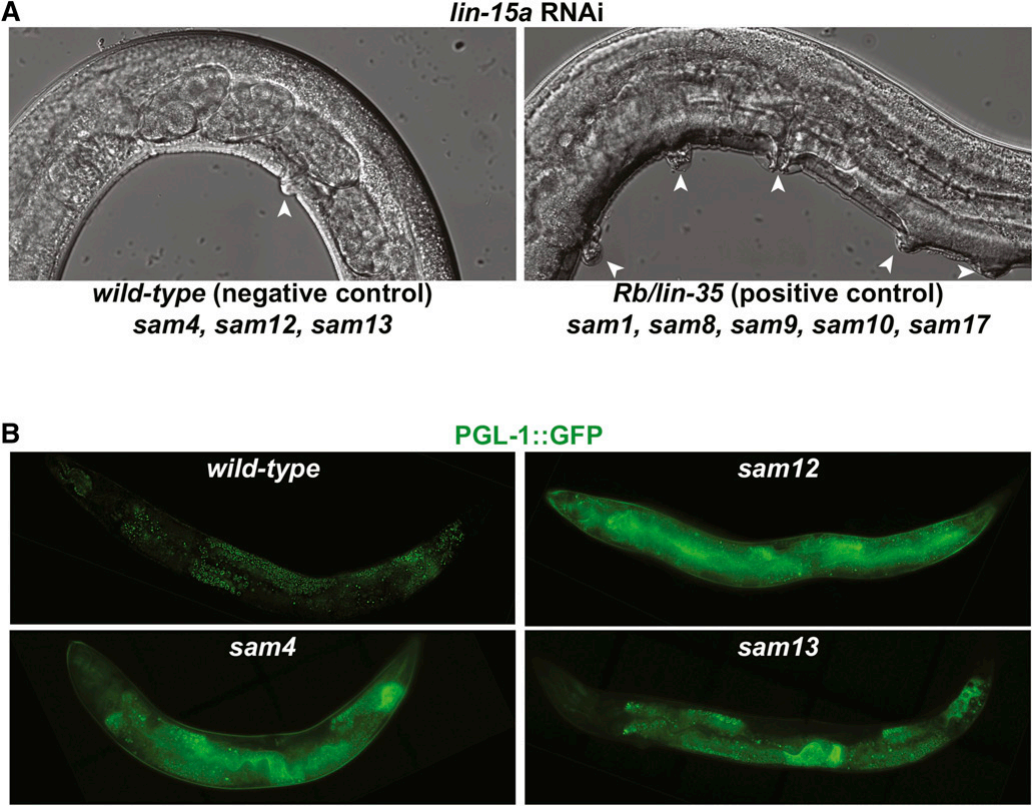
Characterization of mutant alleles. (A) Images of wild-type and *lin-35* mutant worms fed *lin-15a* RNAi. Arrowheads point to vulvae. (B) Fluorescent images of PGL-1::GFP expressed in the germline (wild-type), intestine (*sam4* and *sam13*), and throughout the soma (*sam12*).

Most components of the synMuv B heterochromatin complex antagonize P-granule accumulation in somatic cells (Petrella *et al.* 2011). In *C. elegans*, components of this pathway are also known as synMuv B genes because they exhibit a synthetic (syn) multi-vulva (Muv) phenotype when combined with a mutation in a separate synMuv A class of genes. The class A synMuvs do not exhibit somatic P granules. Because screens for synMuv B mutants looking for the Muv phenotype have been completed to near saturation, we sought to distinguish mutations in the synMuv B pathway from those in a novel pathway. To do this, we used an RNAi feeding vector to knockdown expression of the synMuv A gene *lin-15a* in all eight of the new alleles. synMuv B mutants fed *lin-15a* RNAi exhibit a fully penetrant multi-vulva phenotype (Bosher *et al.* 1999), which can be observed in the *lin-35*/Rb mutant (positive synMuv B control) but not in wild-type worms (Figure 2A, arrowheads mark vulvae). We repeated *lin-15a* RNAi in triplicate and found that *sam1*, *sam8*, *sam9*, *sam10*, and *sam17* fall into the synMuv B class of mutants (3/3 replicates), validating the specificity of our screen (Table 1). However, *sam4*, *sam12*, and *sam13* alleles did not exhibit multiple vulvae after *lin-15a* RNAi (0/3 replicates), making them likely to contain mutations in genes that act in parallel or downstream of the synMuv B pathway.

Somatic PGL-1::GFP expression in *sam4* and *sam13* mutants is restricted to intestinal cells, whereas *sam12* mutants express PGL-1 granules throughout the worm (Figure 2B). When these strains are backcrossed into the wild-type PGL-1::GFP parental strain, F1 progeny no longer express somatic PGL-1 granules. This suggests that each of these mutants are recessive for the somatic PGL-1::GFP-granule phenotype and are likely loss-of-function alleles.

**Figure 3:**
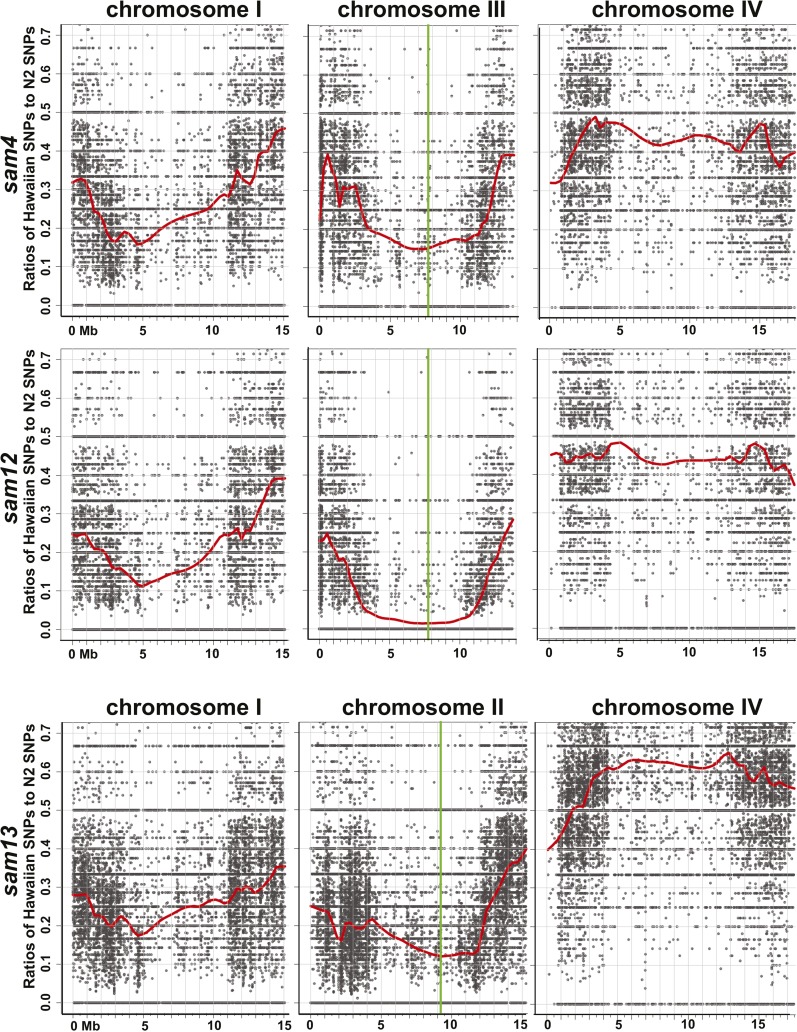
Hawaiian Variant Mapping and CloudMap analysis. Ratios of Hawaiian to N2 SNPs show linkage in the center of chromosome III for *sam4* and *sam12*, and linkage on chromosome II for *sam13*. Green vertical bars indicate the position of *lin-13* on III and of *dpl-1* on II. Even after SNP normalization, some degree of linkage was observed at 5 Mb on chromosome I for all three mutants. Chromosome IV, which is unlinked, is shown for comparison.

To identify genetic lesions in the *sam4*, *sam12*, and *sam13* alleles, Hawaiian Variant Mapping was used in combination with genome-wide sequencing, and mutations were identified using the CloudMap pipeline (Minevich *et al.* 2012). Linkage to somatic PGL-1::GFP was observed on chromosome II for *sam13*, and on chromosome III for *sam4* and *sam12* (Figure 3). We also observed some linkage to chromosome I at approximately 5 Mb for all three alleles, which most likely reflects the integration site of the PGL-1::GFP transgene that maps to chromosome I.

**Figure 4:**
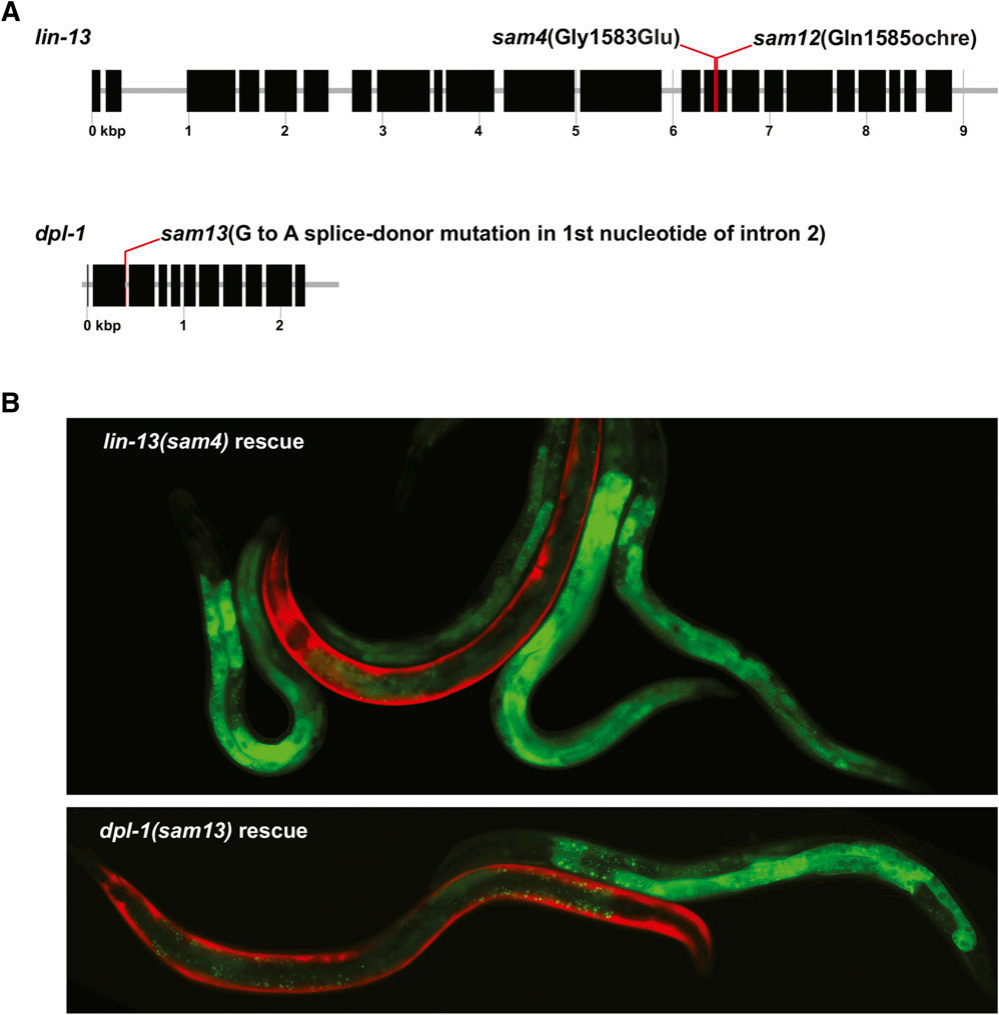
Position and rescue of *lin-13* and *dpl-1* mutations. (A) *sam4* and *sam12* mutations are both located in exon 14 of *lin-13*. *sam13* is a splice-donor mutation in the first nucleotide of intron 2 in *dpl-1*. (B) Transgenic *sam4* (top) and *sam13* (bottom) worms rescued by fosmids carrying *lin-13* or *dpl-1* (transgenes marked with a red co-expression marker). Loss of transgenes cause somatic PGL-1::GFP re-expression.

On chromosome III, we found four nonsynonymous mutations closely linked to *sam4*, one of which contained a G to A mutation that causes a G1583E substitution in LIN-13 (Figure 4A). RNAi depletion of the three other genes carrying nonsynonymous mutations did not cause somatic P-granule expression (data not shown). We generated a line with a fosmid containing *lin-13* (marked with a *myo-3*::mCherry transgene) (Figure 4B) and observed rescue in 14 of 20 L4-staged worms carrying the transgene; 28/28 siblings without the transgene re-expressed intestinal PGL-1::GFP, suggesting that the amino acid substitution in LIN-13 is responsible for the phenotype. LIN-13 is a known *lin-35*/Rb pathway component that binds and recruits the heterochromatin protein HPL-2 to distinct nuclear foci (Meléndez and Greenwald 2000; Coustham *et al.* 2006) and also acts with LIN-35 and HPL-2 to dampen the ER stress response (Kozlowski *et al.* 2014). In addition to being a known synMuv B gene, *lin-13* mutants were previously shown to exhibit somatic P-granule expression (Wang *et al.* 2005), suggesting that *sam4* could be a hypomorphic allele of *lin-13* that is not strong enough to cause a synMuv phenotype when combined with *lin-15a* RNAi. Despite *sam4* and *sam12* exhibiting different degrees of somatic P-granule expression, these two alleles failed to complement, suggesting that they contain mutations in the same gene (Table 1). *sam4/sam12* cross progeny displayed an intermediate phenotype (100/100 cross progeny with somatic PGL-1::GFP expression), suggesting that *sam12* represents a stronger loss-of-function when compared to *sam4* in an allelic series. On chromosome III, whole genome sequencing found four nonsynonymous mutations and one stop-gained mutation closely linked to *sam12*; however, RNAi depletion of these genes did not cause somatic P-granule expression (not shown). Sequence coverage was low and incomplete across *lin-13* in *sam12* worms, so we sequenced *lin-13* and found a C to T mutation that introduced a stop codon (Q1585*) (Figure 4A). Low broods (∼10/worm) in *lin-13(sam12)* mutants prevented us from obtaining transgenic lines to test rescue. The predicted *lin-13(n387)* null allele is homozygous sterile in the absence of maternal LIN-13 (Meléndez and Greenwald 2000). Although *lin-13(sam12)* worms exhibit very slow growth and low broods, *sam12* is an unlikely null because it is possible to maintain homozygous mutants.

On chromosome II, we found one splice site donor, three frameshift, and three nonsynonymous mutations closely linked to *sam13*. The splice-donor mutation is a G to A base pair substitution in the first nucleotide of intron 2 in *dpl-1* (Figure 4A). Of the seven genes mutated in *sam13*, only *dpl-1* RNAi causes somatic P-granule expression. This result was anticipated as DPL-1, also a synMuv B protein in the LIN-35/Rb complex, was previously reported to repress somatic P-granule expression (Ceol and Horvitz 2001; Wang *et al.* 2005). We generated a line with a fosmid containing *dpl-1* (Figure 4B); 16/16 progeny with the transgene rescued, whereas 32/32 siblings without the transgene re-expressed intestinal PGL-1::GFP, suggesting the splice-donor mutation in *dpl-1* is responsible for the phenotype. DPL-1 encodes a homolog of human DP, the heterodimerization partner of the E2F transcription factor (Ceol and Horvitz 2001). In *C. elegans*, DPL-1 initiates spermatheca dilation to promote ovulation and fertilization, and strong loss-of-function mutations inhibit ovulation and oocytes undergo endomitosis (Chi and Reinke 2006, 2009). *dpl-1(sam13)* animals do not appear to have defects in ovulation, suggesting that this allele causes a splicing defect that only weakly compromises DPL-1 function.

**Figure 5:**
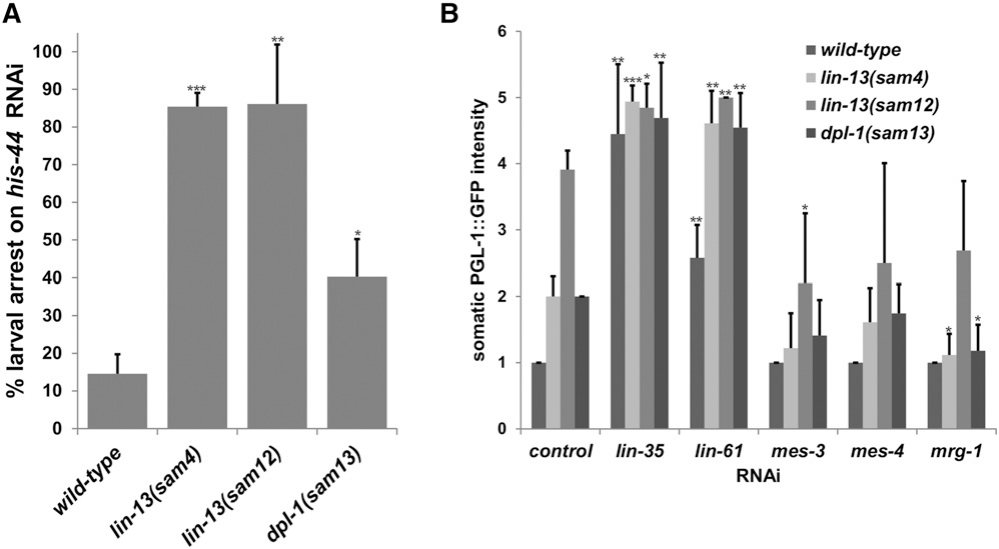
RNAi enhancement and PGL-1::GFP suppression. (A) *sam4*, *sam12*, and *sam13* mutants exhibit increased larval arrest on *his-44* RNAi. (B) Somatic PGL-1::GFP intensity is enhanced with *lin-35* and *lin-61* RNAi, but partially suppressed with *mes-3*, *mes-4*, and *mrg-1* RNAi. Error bars = SD. p values: ***<0.0005, **<0.005, and *<0.05.

The two *lin-13* alleles and the splice site donor mutation in *dpl-1* are not synMuv with *lin-15a* RNAi at 20°, so we asked if these alleles are RNAi defective or whether they demonstrate enhanced RNAi sensitivity associated with known *lin-13*, *dpl-1*, and other synMuv B mutants. The presence of P granules makes the *C. elegans* germline exceptionally sensitive to RNAi, and germline RNAi is defective when P-granule assembly is compromised in the absence of PGL-1 (Robert *et al.* 2005). Somatic P granules in synMuv B mutants cause enhanced RNAi sensitivity throughout the body of the worm (Wang *et al.* 2005). To determine if *sam4*, *sam12*, and *sam13* exhibit enhanced or defective RNAi sensitivity, we performed feeding RNAi on *his-44*. *his-44* RNAi feeding has been shown to cause only 12% early larval arrest in wild-type worms, but it causes 86% arrest in the *rrf-3(pk1426)* RNAi sensitive strain (Wang and Ruvkun 2004). Like other synMuv B mutants, all three alleles showed enhanced larval arrest on *his-44* RNAi (Figure 5A), suggesting that the absence of a synMuv phenotype with *lin-15a* RNAi cannot be attributed to defective RNAi. Both Rb/*lin-35* and MBT/*lin-61* RNAi further enhanced somatic P-granule expression in all three alleles, suggesting that they are hypomorphic, and not amorphic, alleles (Figure 5B). Somatic P-granule expression is suppressed in known synMuv B mutants by RNAi depletion of the chromatin regulators *mes-3*, *mes-4*, and *mrg-1* (Unhavaithaya *et al.* 2002; Wang *et al.* 2005; Cui *et al.* 2006; Andersen *et al.* 2006; Takasaki *et al.* 2007; Rechtsteiner *et al.* 2010). We also observed mild suppression when these chromatin regulators were depleted in *sam4*, *sam12*, and *sam13* mutants (Figure 5B). Some alleles of *lin-13* have been shown to exhibit a Muv phenotype independent of synMuv A at elevated temperatures (Ferguson and Horvitz 1985), so *lin-15a* RNAi was performed on *lin-13(sam4)*, *lin-13(sam12)*, and *dpl-1(sam13)* mutants at 25°. At this temperature, Muv animals were still not observed with control or *lin-15a* RNAi on *lin-13(sam4)* and *lin-13(sam12)* worms (0/3 replicates). However, at 25° Muv worms were observed on 3/3 replicates of *dpl-1(sam13)* worms fed *lin-15a* RNAi (average 39.9% Muv; SD = 4.5%), but not control RNAi (0/3 replicates). Collectively, these results suggest that *sam4*, *sam12*, and *sam13* share features with previously described loss-of-function *lin-13* and *dpl-1* alleles, with the exception that they do not readily exhibit synMuv phenotypes.

By screening 4000 haploid genomes, we expected to get one to two loss-of-function mutations of each gene required to suppress the somatic expression of P granules (Jorgensen and Mango 2002). Of the eight mutations we obtained from the screen, only synMuv B genes were isolated. Because this screen was not performed to saturation, it does not exclude the possibility that non-synMuv B components repress the somatic expression of germline programs, but it does suggest that if they exist, then they are either rare or essential for early development. Notably, we did not recover alleles of *daf-2*, a gene whose depletion was previously shown to cause ectopic expression of GFP::PGL-1 driven behind a *pie-1* promoter (Curran *et al.* 2009). This may be due to the limited number of haploid genomes screened, or because the PGL-1::GFP transgene used in our screen is not driven by *pie-1* but rather by the *pgl-1* promoter. Due to the limited scope of this screen, little can be inferred concerning the significance of isolating one *dpl-1* and two *lin-13* alleles instead of other synMuv B genes; however, isolating these alleles may implicate DPL-1 and LIN-13 in a more central role because they do not have to be completely inhibited to cause somatic P-granule expression.

Although it is assumed that germline components expressed in the soma induce germ cell characteristics that could lead to oncogenesis (*i.e.*, pluripotency and the ability to self-renew), it is unclear how germ granules themselves may contribute to this process. Recently, it was discovered that stress granules, another ribonucleoprotein aggregate that shares some components with germ granules, are important for tumor progression; knockdown of the stress granule nucleator G3BP1 reduces local invasive capacity in tumor xenografts (Somasekharan *et al.* 2015). Interestingly, knockdown of the *C. elegans* homolog, *gtbp-1/K08F4.2*, suppresses synMuv A/B phenotypes (Cui *et al.* 2006). Whether stress granules exert protective effects to cancer cells during chemotherapy and radiation or whether stress granules sequester mRNAs encoding factors that inhibit oncogenesis is unknown. However, these findings highlight the importance of translational regulation in cancer and may help to explain how the presence of germline-enriched proteins in tumors can be associated with malignancy and poor patient prognosis.

## References

[bib1] Andersen E. C. Lu X. Horvitz H. R., 2006 C. elegans ISWI and NURF301 antagonize an Rb-like pathway in the determination of multiple cell fates. Development 133: 2695–2704. 16774993 10.1242/dev.02444

[bib2] Blanchard T. Srivastava P. K. Duan F., 2013 Vaccines against advanced melanoma. Clin. Dermatol. 31: 179–190. 23438381 10.1016/j.clindermatol.2012.08.005

[bib3] Bosher J. M. Dufourcq P. Sookhareea S. Labouesse M., 1999 RNA interference can target pre-mRNA: consequences for gene expression in a Caenorhabditis elegans operon. Genetics 153: 1245–1256. 10545456 10.1093/genetics/153.3.1245 PMC1460805

[bib4] Brenner S., 1974 The genetics of Caenorhabditis elegans. Genetics 77: 71–94. 4366476 10.1093/genetics/77.1.71 PMC1213120

[bib5] Campbell A. C. Updike D. L., 2015 CSR-1 and P granules suppress sperm-specific transcription in the C. elegans germline. Development 142: 1745–1755. 25968310 10.1242/dev.121434 PMC4440928

[bib6] Ceol C. J. Horvitz H. R., 2001 dpl-1 DP and efl-1 E2F act with lin-35 Rb to antagonize Ras signaling in C. elegans vulval development. Mol. Cell 7: 461–473. 11463372 10.1016/s1097-2765(01)00194-0

[bib7] Chi W. Reinke V., 2009 DPL-1 (DP) acts in the germ line to coordinate ovulation and fertilization in C. elegans. Mech. Dev. 126: 406–416. 19368797 10.1016/j.mod.2009.01.008 PMC2680456

[bib8] Chi W. Reinke V., 2006 Promotion of oogenesis and embryogenesis in the C. elegans gonad by EFL-1/DPL-1 (E2F) does not require LIN-35 (pRB). Development 133: 3147–3157. 16854972 10.1242/dev.02490

[bib9] Coustham V. Bedet C. Monier K. Schott S. Karali M., 2006 The C. elegans HP1 homologue HPL-2 and the LIN-13 zinc finger protein form a complex implicated in vulval development. Dev. Biol. 297: 308–322. 16890929 10.1016/j.ydbio.2006.04.474

[bib10] Cui M. Kim E. B. Han M., 2006 Diverse chromatin remodeling genes antagonize the Rb-involved SynMuv pathways in C. elegans. PLoS Genet. 2: e74. 16710447 10.1371/journal.pgen.0020074 PMC1463046

[bib11] Curran S. P. Wu X. Riedel C. G. Ruvkun G., 2009 A soma-to-germline transformation in long-lived Caenorhabditis elegans mutants. Nature. 7250: 1079–1084 10.1038/nature08106 PMC2716045 19506556

[bib12] Doitsidou M. Poole R. J. Sarin S. Bigelow H. Hobert O., 2010 C. elegans mutant identification with a one-step whole-genome-sequencing and SNP mapping strategy. PLoS One 5: e15435. 21079745 10.1371/journal.pone.0015435 PMC2975709

[bib13] Ferguson E. L. Horvitz H. R., 1985 Identification and characterization of 22 genes that affect the vulval cell lineages of the nematode Caenorhabditis elegans. Genetics 110: 17–72. 3996896 10.1093/genetics/110.1.17 PMC1202554

[bib14] Frøkjaer-Jensen C. Davis M. W. Hopkins C. E. Newman B. J. Thummel J. M., 2008 Single-copy insertion of transgenes in Caenorhabditis elegans. Nat. Genet. 40: 1375–1383. 18953339 10.1038/ng.248 PMC2749959

[bib15] Georlette D. Ahn S. MacAlpine D. M. Cheung E. Lewis P. W., 2007 Genomic profiling and expression studies reveal both positive and negative activities for the Drosophila Myb MuvB/dREAM complex in proliferating cells. Genes Dev. 21: 2880–2896. 17978103 10.1101/gad.1600107 PMC2049191

[bib16] Janic A. Mendizabal L. Llamazares S. Rossell D. Gonzalez C., 2010 Ectopic expression of germline genes drives malignant brain tumor growth in Drosophila. Science 330: 1824–1827. 21205669 10.1126/science.1195481

[bib17] Jorgensen E. M. Mango S. E., 2002 The art and design of genetic screens: caenorhabditis elegans. Nat. Rev. Genet. 3: 356–369. 11988761 10.1038/nrg794

[bib18] Kamath R. S. Fraser A. G. Dong Y. Poulin G. Durbin R., 2003 Systematic functional analysis of the Caenorhabditis elegans genome using RNAi. Nature 421: 231–237. 12529635 10.1038/nature01278

[bib19] Kozlowski L. Garvis S. Bedet C. Palladino F., 2014 The Caenorhabditis elegans HP1 family protein HPL-2 maintains ER homeostasis through the UPR and hormesis. Proc. Natl. Acad. Sci. USA 111: 5956–5961. 24715729 10.1073/pnas.1321698111 PMC4000850

[bib20] Kutscher, L. M., and S. Shaham, 2014 Forward and reverse mutagenesis in C. elegans. WormBook January 17: 1–26. 10.1895/wormbook.1.167.1 PMC4078664 24449699

[bib21] Meléndez A. Greenwald I., 2000 Caenorhabditis elegans lin-13, a member of the LIN-35 Rb class of genes involved in vulval development, encodes a protein with zinc fingers and an LXCXE motif. Genetics 155: 1127–1137. 10880475 10.1093/genetics/155.3.1127 PMC1461134

[bib22] Minevich G. Park D. S. Blankenberg D. Poole R. J. Hobert O., 2012 CloudMap: a cloud-based pipeline for analysis of mutant genome sequences. Genetics 192: 1249–1269. 23051646 10.1534/genetics.112.144204 PMC3512137

[bib23] Petrella L. N. Wang W., C. A. Spike, A. Rechtsteiner, V. Reinke *et al*, 2011 synMuv B proteins antagonize germline fate in the intestine and ensure C. elegans survival. Development 138: 1069–1079. 21343362 10.1242/dev.059501 PMC3042865

[bib24] Rechtsteiner A. Ercan S. Takasaki T. Phippen T. M. Egelhofer T. A., 2010 The histone H3K36 methyltransferase MES-4 acts epigenetically to transmit the memory of germline gene expression to progeny. PLoS Genet. 6: e1001091. 20824077 10.1371/journal.pgen.1001091 PMC2932692

[bib25] Robert V. J. Sijen T. van Wolfswinkel J. Plasterk R. H., 2005 Chromatin and RNAi factors protect the C. elegans germline against repetitive sequences. Genes Dev. 19: 782–787. 15774721 10.1101/gad.332305 PMC1074315

[bib26] Sahin U. Türeci O. Chen Y. T. Seitz G. Villena-Heinsen C., 1998 Expression of multiple cancer/testis (CT) antigens in breast cancer and melanoma: basis for polyvalent CT vaccine strategies. Int. J. Cancer 78: 387–389. 9766577 10.1002/(SICI)1097-0215(19981029)78:3<387::AID-IJC22>3.0.CO;2-2

[bib27] Simpson A. J. G. Caballero O. L. Jungbluth A. Chen Y.-T. Old L. J., 2005 Cancer/testis antigens, gametogenesis and cancer. Nat. Rev. Cancer 5: 615–625. 16034368 10.1038/nrc1669

[bib28] Somasekharan S. P. El-Naggar A. Leprivier G. Cheng H. Hajee S., 2015 YB-1 regulates stress granule formation and tumor progression by translationally activating G3BP1. J. Cell Biol. 208: 913–929. 25800057 10.1083/jcb.201411047 PMC4384734

[bib29] Strome S. Updike D. L., 2015 Specifying and protecting germ cell fate. Nat. Rev. Mol. Cell Biol. 16: 406–416. 26122616 10.1038/nrm4009 PMC4698964

[bib30] Takasaki T. Liu Z. Habara Y. Nishiwaki K. Nakayama J.-I., 2007 MRG-1, an autosome-associated protein, silences X-linked genes and protects germline immortality in Caenorhabditis elegans. Development 134: 757–767. 17215300 10.1242/dev.02771 PMC2435364

[bib31] Unhavaithaya Y. Shin T. H. Miliaras N. Lee J. Oyama T., 2002 MEP-1 and a homolog of the NURD complex component Mi-2 act together to maintain germline-soma distinctions in C. elegans. Cell 111: 991–1002. 12507426 10.1016/s0092-8674(02)01202-3

[bib32] Updike D. L. Knutson A. K. Egelhofer T. A. Campbell A. C. Strome S., 2014 Germ-granule components prevent somatic development in the C. elegans germline. Curr. Biol. 24: 970–975. 24746798 10.1016/j.cub.2014.03.015 PMC4036631

[bib33] Updike D. L. Strome S., 2009 A genomewide RNAi screen for genes that affect the stability, distribution and function of P granules in Caenorhabditis elegans. Genetics 183: 1397–1419. 19805813 10.1534/genetics.109.110171 PMC2787428

[bib34] Wang D. Kennedy S. Conte D. Jr Kim J. K. Gabel H. W., 2005 Somatic misexpression of germline P granules and enhanced RNA interference in retinoblastoma pathway mutants. Nature 436: 593–597. 16049496 10.1038/nature04010

[bib35] Wang D. Ruvkun G., 2004 Regulation of Caenorhabditis elegans RNA interference by the daf-2 insulin stress and longevity signaling pathway. Cold Spring Harb. Symp. Quant. Biol. 69: 429–431. 16117677 10.1101/sqb.2004.69.429

[bib36] Whitehurst A. W., 2013 Cause and consequence of cancer/testis antigen activation in cancer. Annu. Rev. Pharmacol. Toxicol. 54: 1–22. 24160706 10.1146/annurev-pharmtox-011112-140326

[bib37] Wu X. Shi Z. Cui M. Han M. Ruvkun G., 2012 Repression of germline RNAi pathways in somatic cells by retinoblastoma pathway chromatin complexes. PLoS Genet. 8: e1002542. 22412383 10.1371/journal.pgen.1002542 PMC3297578

